# Use of Selected Environmental Lactic Acid Bacteria During Industrial Production of Heat-Treated Nitrite-Free Organic Sausage

**DOI:** 10.3390/foods14061028

**Published:** 2025-03-18

**Authors:** Piotr Szymański, Anna Okoń, Dorota Zielińska, Beata Łaszkiewicz, Danuta Kołożyn-Krajewska, Zbigniew J. Dolatowski

**Affiliations:** 1 Department of Meat and Fat Technology, Prof. Waclaw Dabrowski Institute of Agriculture and Food Biotechnology—State Research Institute (IBPRS-PIB), 36 Rakowiecka Street, 02-532 Warsaw, Poland; anna.okon@ibprs.pl (A.O.); beata.laszkiewicz@ibprs.pl (B.Ł.); zbigniew.dolatowski@ibprs.pl (Z.J.D.); 2 Institute of Human Nutrition Sciences, Warsaw University of Life Sciences (WULS-SGGW), 166 Nowoursynowska Street, 02-787 Warsaw, Poland; dorota_zielinska@sggw.edu.pl; 3 Department of Dietetics and Food Studies, Faculty of Science and Technology, Jan Dlugosz University (UJD), 13/15 Armii Krajowej Avenue, 42-200 Czestochowa, Poland; d.kolozyn-krajewska@ujd.edu.pl

**Keywords:** novel technology, nitrite-free sausages, *Lactiplantibacillus plantarum* S21, acid whey, organic products, quality

## Abstract

This study aimed to evaluate the potential of lactic acid bacteria (LAB) isolated from organic acid whey as an alternative to nitrites in heat-treated organic sausages. Eleven LAB strains were screened for their ability to develop sensory characteristics similar to traditionally cured meat. Based on the results, *Lactiplantibacillus plantarum* S21 was selected for further experiments. Four sausage treatments were produced: control cured (C), salted (S), salted with *L. plantarum* S21 at 10^7^ CFU/g (LP), and salted with acid whey (AW). The pH value, oxidation-reduction potential (ORP), antioxidant activity of peptides (ABTS•+), thiobarbituric acid-reactive substance (TBARS), fatty acid profile, and microbiological quality were assessed post-production and after 14 days of cold storage. After production, the LP and AW sausages had a lower pH than the cured (C) and uncured (S) control samples. LP sausages exhibited a stable pink colour due to myoglobin conversion to nitrosylmyoglobin, comparable to the cured control. The LP sausages were similar in overall sensory quality to the cured (C) samples and were superior to the S and AW sausages after storage. The lowest ORP value was observed in treatment C after production, whereas after storage, no significant differences were found between the treatments. The highest antioxidant activity of peptides was observed in the LP sausages. It was shown that the LP and AW treatments had lower saturated fatty acid content and higher monounsaturated and polyunsaturated fatty acid content than the C and S treatments. Nevertheless, the C treatment had the lowest TBARS value. Lower total viable counts were found in the C and LP treatments than in the S and AW treatments after storage. Our research demonstrates the potential of *L. plantarum* S21 for producing heat-treated sausages without nitrites, assuming the implementation of additional anti-botulinum barriers. Nevertheless, further studies on the role of bacteria in meat oxidation processes are needed.

## 1. Introduction

Consumer interest in traditional and organic food, free from synthetic additives, has significantly increased in recent years [1]. However, organic meat processing poses significant challenges, particularly due to legal restrictions on using chemical food additives, such as nitrites, which play a crucial role in conventional meat curing [2]. Nitrites are widely used to develop the characteristic sensory properties of cured meat products, including colour, flavour, and aroma. Additionally, they contribute to oxidative stability and inhibit the proliferation of harmful microorganisms such as *Clostridium botulinum* [3,4,5]. However, nitrites can also react with secondary amines from protein and lipid degradation, forming N-nitrosamines (NAs), which have been linked to carcinogenic effects [6]. The European Food Safety Authority (EFSA) has reported that dietary nitrite intake from direct additives and environmental contamination may increase overall exposure to NAs [7]. As a result, efforts to reduce or eliminate nitrites in meat processing have been a longstanding research focus. Vitamins, plant extracts, spices, herbs, and fruits have all been studied as potential alternatives, as these substances or their components demonstrated antioxidant and/or bacteriostatic effects [5,8,9]. Some vegetable and fruit extracts were also tested for their meat colouring properties [10]. Another method that has already been industrialised involves replacing nitrites with vegetable juices or extracts containing naturally occurring nitrates while simultaneously introducing a bacterial culture to meat capable of reducing NO_3_ to NO_2_ [11]. Nevertheless, in this technique, the source of nitrites is altered rather than eliminated. With the partial replacement of nitrites, high hydrostatic pressure (HHP) can also be applied to extend the shelf life of meat products [5]. Despite numerous studies, no comprehensive replacement has been found that replicates all functional properties of nitrites. Moreover, the complete elimination of nitrite poses a challenge in achieving the characteristic desirable colour and flavour of cured meat.

A promising avenue in food bioscience involves harnessing beneficial microorganisms, particularly lactic acid bacteria (LAB), from naturally fermented products and environmental niches. Certain LAB strains exhibit enzymatic activities that may contribute to the development of key meat product characteristics without nitrite. For instance, some LAB can convert myoglobin (MbFe^2+^) into nitrosylmyoglobin (MbFe^2+^NO), imparting the desirable pink colour without the need for synthetic nitrites [12,13,14]. Some strains exhibit antioxidant properties, which are associated with the activity of specific bacterial enzymes, such as glutathione reductase (GR) and glutathione peroxidase (GPx) [15]. LAB antibacterial properties are associated with formation of substances that are the products of bacterial metabolic pathways, including lactic acid, acetic acid, pyruvic acid, and bacteriocins [16,17]. As research indicates, these biochemical properties of bacteria can be utilised to reduce the amount of nitrite added to meat [18,19]. Certain studies indicated that some strains of LAB may also impact the curing flavour of meat products [20].

Interesting research findings on the replacement of nitrites in meat products have been obtained through the use of organic acid whey. The application of this process has been demonstrated to exert a beneficial influence on the colour, flavour, and durability of uncured meat products [21,22,23]. Also, our recent research shows that LAB derived from acid whey have demonstrated the ability to generate MbFe^2+^NO, producing a stable pink colour in uncured meat products for up to 56 days under cold storage conditions [24]. A potential mechanism for the formation of the pink pigment in meat is the synthesis of NO with the contribution of *L. fermentum* S8 from the nitrogen residue of L-arginine in the attendance of NADPH coenzyme and oxygen with the involvement of NOS; then, the reaction between NO and MbFe^2+^ occurs and leads to the formation of MbFe^2+^NO [13,24]. Although these results are encouraging regarding the use of lactic acid bacteria from acid whey to develop a pink colour in meat without the addition of nitrites, further research is needed. It is crucial to assess to what extent the bacteria, and which ones, can replicate the multifunctional role of nitrites in meat processing, including their contribution to flavour development as well as their antimicrobial and antioxidant properties.

To sum up, there is documented evidence of the need to search for alternatives to using nitrites in meat processing, both organic (due to legal requirements) and conventional, for health reasons. This problem became the basis of our research.

The study aimed to evaluate the potential of selected lactic acid bacteria isolated from organic acid whey as an alternative to nitrites in heat-treated organic sausages. This study assessed the impact of these bacteria on the physicochemical, oxidative, fatty acid profile, sensory, and microbiological properties of uncured, heat-treated, organic sausages. A treatment with organic acid whey was also included for comparison.

The novelty of the presented study was the developed nitrite-free organic sausage technology that uses the environmental strains of bacteria from acid whey and applies the new technology under industrial conditions.

## 2. Materials and Methods

### 2.1. LAB Strains and Culture Conditions

The 11 LAB strains derived from the microbial collection belonging to the Chair of Food Hygiene and Quality Management at WULS-SGGW were employed in this study (Table 1). The strains were originally isolated from organic acid whey and subsequently identified genetically. Their safety, biochemical, and enzyme activity were also tested in the study of Rzepkowska et al. [17]. Briefly, the lactobacilli strains represented an appropriate antibiotic sensitivity. They do not possess any transfer resistance genes and do not produce harmful enzymes and biogenic amines. Moreover, the strains possess good antimicrobial activity. The LAB strains were prepared using the protocol described in previous studies [24]. The combination of bacterial strains with a 0.85% sodium chloride solution was incorporated into the meat mixture, resulting in a bacterial concentration of approximately 10^7^ CFU per gram of meat.

### 2.2. Preparation of Acid Whey

The acid whey from unpasteurised organic cottage cheese produced on a Polish farmyard Jasiołka Meat Plant (Dukla, Poland) in the Podkarpacie region was used in the study (Cert. No. PL-EKO-05.616-0007076.2023.001). The average values of the parameters pH and colour of the acid whey reached 4.65 and L* = 33.85, a* = −1.95 and b* = 0.39, respectively. The total viable count in the acid whey was 5.69 CFU/mL, and the LAB concentration was 5.28 log CFU/mL. The pathogenic bacteria *Salmonella* spp., *S. aureus*, and *L. monocytogenes* were not detected in the acid whey.

### 2.3. Raw Meat Materials and Additives

The raw material for the study was organic meat pork from pigs from an organic farm (Cert. No. PL-EKO-01.616-0000505.2023.002): ham muscles (*M. semimembranosus, M. semitendinosus*, and *M. biceps femoris*) and fatty meat from ham (trimmings, fat content 40%) processed at a medium-sized meat-processing plant Jasiołka Meat Plant, Dukla, Poland (Cert. No. PL-EKO-01.616-0009988.2023.001). The meat was excised at 48 h post mortem from carcasses cooled down at 2 °C. The pH of the muscles was between 5.5 and 6.1. Spices and glucose came from organic producers.

### 2.4. The Experiment Scheme

**Figure 1:**
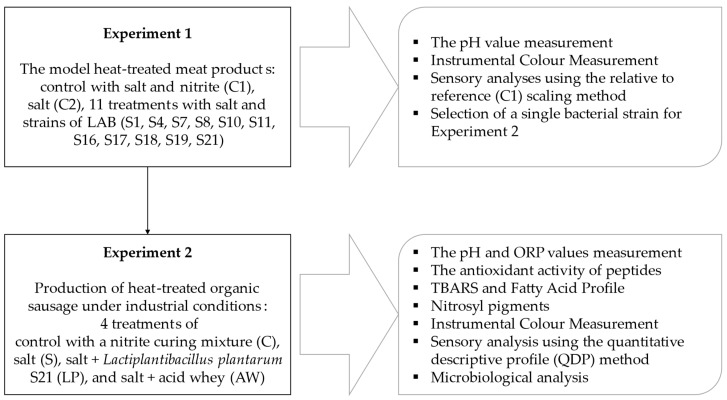
The scheme of experiments.

The experimental design is presented in Figure 1. The study was divided into two stages that are logically interconnected.

### 2.5. The Model Meat Product (Experiment 1)

Thirteen meat batter treatments were prepared (Table 2): control treatment with salt and NaNO_2_ at 100 mg/kg (C1), treatment with salt (C2), and 11 treatments with salt and strains of lactic acid bacteria in count 10^7^ CFU/g (S1, S4, S7, S8, S10, S11, S16, S17, S18, S19, S21). The minced meat (3 mm diameter mesh) was combined with the other ingredients using a mixer (Keripar, Troy, OH, USA). The bacterial strains were added separately, according to the specific treatment (S1, S4, S7, S8, S10, S11, S16, S17, S18, S19, S21). The meat batter was canned using a vacuum stuffer (Robot 500, Vemag Maschinenbau GmbH, Verden, Germany) into 190 g cans.

The cans were kept at 4–6 °C for 48 h and then cooked in a cooking kettle (B-type, Brokelmann, Ense-Höingen, Germany). The cans were pre-cooked to achieve the internal temperature of 40 °C for four hours to ensure the strains remained active according to Szymański [24]. Subsequently, the cooking process was continued until the meat temperature reached 70 °C. Then, the cans were cooled to 4–6 °C. The temperature within the cans was continuously monitored with thermocouples (Ellab ctf84, Ellab Validation & Monitoring Solutions, Hillerød, Denmark).

The model meat production process was conducted on three replications (n = 3) within the facilities of the Department of Meat and Fat Technology (IBPRS-PIB, Warsaw, Poland), utilising three distinct lots of pork meat (batches). A total of 1014 cans were produced (13 treatments × 26 cans × 3 replications). After production, the following analyses were carried out: the colour parameters (L*, a*, b*, h*, C*), pH, and sensory evaluation.

This research phase aimed at selecting LAB strains isolated from organic acid whey, which influence the development of the pink colour and the formation of sensory features (flavour, odour, consistency, and overall appearance) most similar to typical cured meat products. Based on these criteria, a single LAB strain was selected for utilisation in Experiment 2 (Section 2.6).

### 2.6. Production of Heat-Treated Organic Sausage Under Industrial Conditions (Experiment 2)

Four sausage treatments were prepared (Table 3): the control treatment with nitrite curing mixture (C), treatment with only salt (S), treatment with salt and *Lactiplantibacillus plantarum* S21 at approx. 10^7^ CFU/g (LP), and treatment with salt and acid whey (5%) (AW).

The minced ham muscles (20 mm diameter mesh) and trimming meat (5 mm diameter mesh) were combined with other ingredients without spices and water.

The meat batters were placed in 48 L polypropylene containers and left to rest for 48 h at 4–6 °C. Next, water/ice and spices were added to the meat batters, mixed and stuffed into 55 mm collagen casings.

The sausages were clipped to a length of 35 cm. Following the stuffing process, the sausages were left to settle for six hours at 20–25 °C. Subsequently, the sausages were dried with hot air (50 °C) for 1 h in the chamber and traditionally smoked (using beech wood billet) with the application of hot (55–65 °C) smoke for 1 h. Thereafter, the sausages were subjected to a heat treatment with hot air (100 °C) up to 70 °C in the centre of the sausage links. The product was subsequently cooled to 15–20 °C with the aid of water, then further cooled to 4–6 °C with cold air in a refrigerated storage facility. The weight loss during the thermal processing of sausages was approx. 20%.

The final product was divided into three separate portions, each vacuum-packed and kept at 4–6 °C. The production procedure was repeated on three occasions, utilising three distinct batches of pork, within the facilities of the ecological meat-processing plant (Cert. No. PL-EKO-01.616-0009988.2023.001).

The sausages were subjected to analysis at three distinct time points: immediately after production (0 days) and after 7 and 14 days of cold storage. The following analyses were carried out: pH, ORP, TBARS, MbFe^2+^NO content, colour parameters (L*, a*, b*, h*, C*, ΔE*), and microbiological. Fatty acid composition and sensory evaluation were performed after production (0 days) and 14 days of storage. ABTS•+ values were analysed after production.

### 2.7. Measurement of pH and Oxidation-Reduction Potential (ORP)

The pH and ORP levels were determined in the meat products following the method described by Okoń et al. [25]. The pH measurements were conducted using a Mettler Toledo pH meter Delta 350 Mettler Toledo, Greifensee, Switzerland), which is equipped with an integrated glass body pH electrode (Mettler Toledo, Greifensee, Switzerland). Temperature compensation has been performed. The ORP measurements were performed with a Mettler Toledo combined electrode InLab Redox with a platinum ring (Mettler Toledo, Greifensee, Switzerland).

### 2.8. Determination of the Antioxidant Activity of Peptides (ABTS•+ Scavenging Assay)

The ABTS•+ (2-Azino-bis-3-ethylbenzothiazoline-6-sulfonic acid) was quantified using the ABTS radical cation decolourisation assay described by Re et al. [26]. The absorbance at 734 nm was determined using a UV–visible U-2900 Hitachi spectrophotometer (Hitachi High-Tech, Tokyo, Japan). The results were expressed as the scavenging ability of the peptides towards ABTS radical cations, with the data presented as Trolox equivalents in mM relative to the peptide concentration (mg). The peptide concentration was calculated according to [27,28]. The U-2900 UV–visible Hitachi spectrophotometer (Hitachi High-Tech, Tokyo, Japan) was also used to measure the absorbances at 750 nm against the blank.

### 2.9. Thiobarbituric Acid-Reactive Substance (TBARS)

The TBARS index was determined using the method of Pikul et al. [29]. A mixture of 10 g of each sample, 34.25 mL of 4% cold perchloric acid, and 750 µL of 0.01% ethanolic BHT solution was homogenised for 1 min using a blender (Bamix m200, ESGE AG, Hauptstrasse 21, CH-9517 Mettlen/Schweiz, Switzerland) and separated on a filter paper. Then, 1 mL of the filtrate was mixed with 1 mL of 0.02 M aqueous 2-thiobarbituric acid solution and incubated at 100 °C for 60 min. Absorbance was measured at 532 nm at approx. 20 °C using a U-2900 spectrophotometer (Hitachi, Tokyo, Japan). A mixture made of 1 mL of 4% cold perchloric acid and 1 mL of 0.02 molar aqueous solution of 2-thiobarbituric acid served as the reference sample. The TBARS values are expressed as mg of malondialdehyde (MDA) per kilogram of the sample.

### 2.10. Fatty Acid Profile

The fatty acid profile was analysed following the [30] protocol, employing gas chromatographic techniques with a flame ion detection system (HP 6890 II-FID; Agilent Technologies, Santa Clara, CA, USA) and a BPX 70 column (Trajan Scientific and Medical, Ringwood, VIC, Australia) with a highly polarised stationary phase. The column parameters were as follows: a length of 100 m, a layer thickness of 0.20 µm, and an internal diameter of 0.25 mm. Internal standard: C21:0 heneicosanoic acid (H5149, Sigma-Aldrich, St Louis, MO, USA). Individual fatty acids were identified based on comparison of retention times with the certified reference material Supelco 37-Component FAME Mix (CRM47885, Merck KGaA, Darmstadt, Germany). The resulting data were expressed as milligrams of fatty acid per 100 g of product.

### 2.11. Nitrosyl Pigments

The nitrosyl pigment levels (MbFe^2+^NO) were extracted from meat samples with an aqueous solution of acetone [31]. The absorbances of the filtrates were read at 540 nm with a spectrophotometer (detailed in Section 2.8). Results were obtained by multiplying the absorbance by 290 and expressed as mg/kg (ppm).

### 2.12. Instrumental Colour Measurement

The Minolta spherical spectrophotometer CR-300 (Konica Minolta, Tokyo, Japan) was used to measure the CIE Lab system colour parameters of the samples (a slice of canned meat or sausage), with L* being the colour lightness, a* the chroma in the range of red and green, and b* the chroma in the range of yellow and blue. The parameters of the measurements were aperture size 8 mm, observation angle 2°, illuminant D65, the default light source was a pulsed xenon lamp, blooming time 5 min, 3 reads per sample, and temperature in the measurement room 24 °C ± 2 °C. The hue angle (h°) and chroma (saturation index) (C*) were calculated using Formulae (1) and (2) with a* and b* being data from an instrumental colour measurement.

(1) $${h{}^{\circ}=\tan}^{-1}\frac{b^{*}}{a^{*}}$$

(2) $$C^{*}=\sqrt{a^{*2}+b^{*2}}$$

Formula (3) was used to calculate the total colour difference value (ΔE*).

(3) $$∆E^{*}=\sqrt{{\left(L1-L2\right)}^{2}+{{\left(a1-a2\right)}^{2}+\left(b1-b2\right)}^{2}}$$

In this, Formula (3) L1, *a*1, *b*1 represents the control treatment, and L2, *a*2, *b*2 represents the colour parameters of other experimental treatments [32,33].

### 2.13. Sensory Analyses

In Experiment 1, the sensory analyses of 12 model meat samples in comparison to reference sample—C1—were performed using the relative-to-reference scaling method [34]. As a tool, a linear 100 mm scale was used anchored identically to reference—completely different. The obtained results were converted into numerical values (0–10 c.u.). For the assessment, the corresponding attributes were used as follows: overall appearance, odour, consistency, and flavour. Each sensory quality feature was assessed on a separate scale. Under the evaluation, the panellists were asked to compare the intensity of selected attributes of a given sample to the reference, which was C1 (the production technology of sample is described in Section 2.5. The model meat product (Experiment 1)). Cans were stored at 24 °C ± 2 °C for 40 min before evaluation. Meat canned product samples were prepared for evaluation by cutting a 50 mm × 30 mm × 20 mm block slice and placing it in 250 mL resealable plastic containers. During the evaluation, the samples were presented in duplicate (evaluated sample + reference sample). One-minute intervals were maintained between assessments. Overall, the evaluations were set as follows: 9 assessors × 6 samples × 2 sessions per day × 2 replications (batches). The same panellists were used across the sessions. Sensory evaluation was carried out after production (time 0).

In Experiment 2, sensory analysis was performed using the quantitative descriptive profile (QDP) method [35]. The QDP is a strict laboratory method in which consumers do not participate. Firstly, meat products were presented to the panellists, and then the descriptors were identified, defined, and verified in a pre-session. To evaluate the sensorial quality of the organic sausage, the six odour discriminators (smoked meat, cured meat, fatty, acid, sharp, and rancid, where 0—not intensive and 10—very intensive), the eight flavour discriminators (smoked meat, cured meat, salty, fatty, acid, bitter, sharp, and rancid, where 0—not intensive and 10—very intensive), and the following traits were estimated: juiciness (0—not very juicy, 10—very juicy), hardness (0—not very hard, 10—very hard), colour (0—light brownish grey, 10—light pink), and overall quality (0—least desirable, 10—most desirable). Meat products were stored at 24 °C ± 2 °C for 40 min before evaluation, and then sausages were prepared for evaluation by cutting two 10 mm thick slices and served in 250 mL resealable plastic containers. The rating and condition of the sensory assessment experiments mode were determined according to Meilgaard et al. [36]. The evaluators received randomly coded and randomly presented samples. Each time, panellists tested samples from all treatments. Overall, the evaluation was set as follows: 9 assessors × 4 samples × 2 sessions × 2 replications (batches). The same panellists were used across the sessions. A sensory assessment of the meat products was performed after production and after 2 weeks of storage.

The assessments (Experiment 1 and Experiment 2) were carried out with the participation of a 9-person (7 women and 2 men, aged 32–62 years) trained panel (according to [37]) assessors of WULS-SGGW, who have over 8–20 years of experience of the sensory evaluation of meat products with various methods and took part in the profiling sessions. The evaluation was conducted in a specially prepared stand during daylight illumination. The meat products were served at room temperature. Water, weak black tea, and crackers were used for mouth cleansing between samples to neutralise the taste and flavour of the meat samples [38]. Ethical approval for the involvement of human subjects in this study was granted by the Rector’s Committee for the Ethics of Scientific Research Involving Humans at WULS-SGGW. Resolution No. 28/RKE/2023/U of 6 July 2023 and informed consent were obtained from all judges prior to initiation of the study.

### 2.14. Microbiological Analysis

To determine the microorganisms, the spread plate technique was used. Nutrient agar (LabM, Heywood, UK), according to [39], was used to calculate the total viable count (TVC), whereas agar MRS (LabM, Bury, UK) was used in the case of LAB enumeration [40]. All the measurements were made in triplicate. The microbial count was presented as the logarithmic value of the colony-forming units per gram (log CFU/g). The enrichment culture methods were used to identify pathogens. The following media were used to determine the presence of Salmonella: XLD agar (Xylose Lysine Deoxycholate Agar, LabM, Heywood, UK) and RAPID’Salmonella agar (Bio-Rad, Hercules, CA, USA) according to ISO method [41]. The presence of *Listeria monocytogenes* was determined using ALOA agar (Bio-Rad, Hercules, CA, USA) and PALCAM agar (LabM, Heywood, UK) [42]. The presence of *Staphylococcus aureus* was identified through the Baird-Parker agar (LabM, Heywood, UK) [43]. The detection of *Clostridium* spp. was conducted according to the standard method [44]. Initially, 0.1 g portions of the sausage samples were transferred into Wrzosek broth (BTL, Łódź, Poland) and incubated under anaerobic conditions at 37 °C for 24–72 h. The positive tubes, as indicated by the turbidity of the broth and gas in the Durham tube, underwent Gram staining. Those tubes containing Gram-positive rods were subsequently inoculated into the Wilson-Blair broth (BTL, Łódź, Poland) and incubated in an anaerobic environment at 37 °C for 24–72 h. These conditions were used to cultivate sulphite-reducing spore anaerobes of the *Clostridium* genus. The results were presented as ‘detected’ or ‘not detected’.

### 2.15. Statistical Analysis

Experiment 1 and Experiment 2 were conducted in three replicates, n = 3 at different times, using three pork meat batches. The colour parameters (L*, a*, b*, h*, C*) and pH values in Experiment 1 were evaluated using one-way ANOVA with a general linear model (GLM), which included the treatments as fixed effects and the replicates as random terms. The results of pH, ORP, TBARS, MbFe^2+^NO content, colour parameters, fatty acid composition, and microbiological obtained from Experiment 2 were analysed by two-way ANOVA with a GLM, which included the treatment and time of storage sausages and their interaction as fixed effects and the replicates as random effects. ABTS•+ values were analysed only for the effect of treatments because these analyses were not repeated over time. For analysing sensory attributes of products in Experiment 1, one-way ANOVA with a general linear model was used. The statistical model includes treatments and panellists as fixed factors and the replicates and sessions as random terms. For analysing sensory attributes of products in Experiment 2, two-way ANOVA (treatments, time of storage) was used with a GLM, which included the treatments, panellists, and treatment × storage time interaction as fixed effects as well as the replicates and sessions as random terms. Fisher’s LSD test was used to determine the significance of the mean values for a multiple comparison at *p* < 0.05. Statistica package, ver.13 (StatSoft Polska Sp. z o.o, Cracow, Poland), was used for analysing the data.

## 3. Results and Discussion

Experiment 1.

### 3.1. The Model Meat Products

#### Colour, Flavour, and Odour Formation

Colour, flavour, and odour are the basic quality attributes of meat products that determine their acceptability by the consumer. The statistical analysis proved a significant impact of the LAB addition on the colour parameters a*, b*, L* (*p* < 0.05). The lowest redness (a* = 8.1) was found in the uncured C2 sample, and the treatments with the addition of LAB (S1, S7, S8, S10, S11, S16, S17, S19, S21) were at significantly higher values of a* parameter (*p* < 0.05), which indicates the bacteria contribution in creating the pink colour of meat without adding sodium nitrite. The highest degree of redness (a* = 9.4) observed in the model products with bacteria was noted in the S21 treatment. The C1 cured control sample exhibited the most significant value of the a* = 12.39 parameter, which was associated with the utilisation of sodium nitrite and the concentration of nitrosomyoglobin in the product [45]. The uncured C2 sample indicated the highest yellowness (b*) and L* parameters among all experimental samples (Table 4), which was attributed to the thermal denaturation of myoglobin to ferrihemochrome and the transformation of colour in treated meat to light grey-brown [46]. Previous experiments demonstrated that meat products manufactured using a bacterial culture of *L. fermentum* S8 at 10^7^ CFU/g of meat have a higher concentration of MbFe^2+^NO, a higher value of a*, and lower values of b* and L* than samples prepared without the addition of bacteria [24]. Luo et al. [13] showed the effect of *L. fermentum* AS1.1880 applied in the amount of 10^7^ CFU/g on the increase in the a* parameter of uncured meat stuffing after incubation. Møller et al. [14] observed an increase in the a* value in fermented uncured sausage with *L. fermentum* JCM1173 at 8 log CFU/g compared to the uncured control sample. Similar observations were made by Møller et al. [14] in an uncured fermented sausage with *L. fermentum* JCM1173 at 10^4^ CFU/g.

Additional insights into the colour characteristics of meat and meat products can be obtained through the measurement of hue angle (h°) and chroma (C*). Statistical analysis of the calculated hue angle (h°) coefficient showed that the S21 treatment had a lower h° value (19.6) than the uncured C2 treatment (h° = 29.6) and other treatments with bacteria added. This is a significant observation, as it indicates a shift in the overall colour hue towards red in the S21 treatment compared to the results of other experimental treatments. The cured treatment (C1) had the significantly lowest h° value (8.1). The S21 treatment also had a relatively high colour saturation (C* = 10.0), most similar to the cured control treatment C1 (C* = 12.5) (Table 4). Higher C* values indicate a more intense and vibrant colouration of the product.

The effects of the treatments were demonstrated on all sensory attributes (flavour, odour, consistency, and overall appearance) of the model meat batters (*p* < 0.05). In the flavour evaluation, treatments S7, S17, and S21 received significantly (*p* < 0.05) the highest scores (6.3–6.9 c.u.), which means that these treatments were closest to the established standard (cured sample C1) in terms of this feature (Table 5). The sensory evaluation showed that sample S7 was the most similar (6.3 c.u.) in terms of odour to the C1 cured standard. The results for the odour determinant that were not statistically different from S7 were also obtained for treatments S21 (5.4 c.u) and S17 (5.4 c.u). In the general appearance, S21 treatment received significantly (*p* < 0.05) the highest scores (5.4 c.u.), which indicates that this sample was the most similar one to the standard C1 of all treatments. The panellists indicated that the colour of meat products was the main feature that distinguished the product in the overall appearance assessment. The obtained results were in line with the colour measurement assay, where the a* values (indicating intensity of red colour) were highest for this sample. The results for consistency for most treatments obtained similar scores (6.0–7.3 c.u.), except for S10 and S19 treatment where the scores were lower (5.3 and 4.8 c.u., respectively). The strains exhibited moderate acidification (pH reduction by an average of 0.25 units relative to the control sample) (Table 4), which is also crucial for the water-holding capacity of meat. It is well documented that LAB can exert a range of effects on the product’s sensory quality. These effects are linked to the bacteria’s metabolic activity and the formation of other organic acids in addition to lactic acid [47,48]. It is commonly known that the palatability profile of cured meat products is determined by the product’s nitrite reaction with proteins, most of all with their functional groups, as well as substances produced as a result of the reaction of nitric oxide as free amino acids and their thiol groups and also with fats [49]. Recent studies also indicate the role of some strains of *Lactiplantibacillus plantarum* in determining the desired flavour and aroma of products with the addition of reduced nitrite [20].

Since the S21 treatment had the highest a* values, the lowest h° values (indicating redness), and the highest C* values in the instrumental evaluation among all tested strains, it was also the most similar to the adopted standard (cured sample C1) in terms of overall appearance in the sensory evaluation. Additionally, it was highly rated for flavour, odour, and consistency. Therefore, the *L. plantarum* S21 strain was selected for further studies and used in Experiment 2.

Experiment 2.

### 3.2. Organic Sausage Production

#### 3.2.1. Analysis of the pH Value

Statistical analysis showed interaction between treatment and storage time for pH value (*p* < 0.05) (Table 6). After production, the LP and AW sausages had lower pH than the cured (C) and uncured (S) control samples (*p* < 0.05), which may be related to the different microbiota of meat stuffing used for producing sausages, their metabolites, and postbiotics after heat treatment including organic acids responsible for the pH of the environment [50,51]. Additionally, in the AW treatment, just simply adding acid whey (pH = 4.65) to the meat could have had an impact on the pH of the product. The dynamics of pH changes during sausage storage depended on the treatment. In the case of treatments C and S, a decrease and subsequent increase in pH were observed during storage, while in the case of treatments LP and AW, a slow increase during storage was observed, although the observed changes were not always statistically significant (Table 6).

The acidity of meat products undergoing cold storage is subject to a number of variables, including the presence and quantity of bacterial metabolites and postbiotics in the product following heat treatment as well as the activity of tissue and bacterial enzymes [18,50,52]. The increased pH value of the product during storage may be attributed to the breakdown of protein by proteases, resulting in the formulation of peptides, as well as amino acids and amines, which serve to neutralise organic acids [53].

#### 3.2.2. Antioxidant Activity of Peptides and Oxidative Stability

Peptides isolated from the organic sausages after production differed significantly (*p* < 0.05) in terms of antioxidant activity concerning the ABTS•+ radical (Table 7).

Significantly the lowest antioxidant activity of peptides was observed in the S sample with salt, and the higher radical scavenging activity of ABTS•+ was reported in the C sample. Similar results were documented by Ferysiuk et al. [54], who observed a statistically significant impact of nitrite quantity on the enhancement of antioxidant activity of peptides towards the ABTS•+ radical in the model canned pork products. Karwowska et al. [55] reported no differences in peptide antioxidant activity between cured and uncured organic pork sausage. In contrast, Wójciak et al. [56] showed lower antioxidant activity of the product towards the ABTS•+ radical in cured roasted beef compared to the uncured sample. Our research indicates the involvement of nitrites in the release of low-molecular protein compounds, but this involvement may be indirect, e.g., by forming the microbiome of meat stuffing [4], which may be involved in proteolysis processes [57]. The significantly higher antioxidant activity of peptides was observed in the LP sample with *L. plantarum* S21 (*p* < 0.05). It is recognised that certain strains of lactic acid bacteria are associated with the synthesis of bioactive peptides that possess antioxidant characteristics [58]. Proteolysis is one of the physiological features of LAB, observed especially in bacteria found in milk. Bacteria need many free amino acids to grow. The proteolytic system of the LAB includes proteinases, peptidases, and specific transport proteins [59]. Depending on the species, subspecies, and even strain, LAB exhibit very diverse proteolytic activity. *Lactiplantibacillus plantarum* shows proteolytic activity, although it is not as high as in *Lacticaseibacillus casei* [60,61]. The mechanism of activity of *L*. *plantarum* S21 may also have been based on the production of acid by bacteria and an increase in meat acidification. The lower pH of the product after thermal treatment was observed in the LP treatment. The increase in acidity may have led to the increased activity of natural proteases (calpain, cathepsin) in the breakdown of the protein substances of muscle tissue into peptides and amino acids, including those with antioxidant features. The AW sample demonstrated the highest antioxidant activity among the peptide samples, with a statistically significant difference (*p* < 0.05) compared to the other samples. Many studies indicate the antioxidant features of whey protein [62,63]. Furthermore, the introduction of acid whey and the activity of environmental LAB resulted in an increase in system acidity, allowing the same mechanism observed in the LP sample to occur. Karwowska et al. [55] showed that uncured sausage cooked with acid whey had higher ABTS•+ radical scavenging activity than the cured sausage. Whereas, in the research by Wójciak et al. [56], it was shown that the roasted beef sample with the acid whey added had lower antioxidant activity of peptides than the control sample without whey, however, the control sample in these studies also had a significantly lower pH.

The redox potential value (ORP) of sausages was determined by treatment and storage time interaction (*p* < 0.01). It was determined that the lowest redox potential value was observed in cured sausage C (317.6 mV) after production. This was attributed to the antioxidant properties of the added sodium nitrite [49,64]. A higher ORP value was found in the S, AW, and LP sausages, where the highest antioxidant activity of the peptides was demonstrated (Table 6). A reduction in redox potential increases the ability to donate electrons and eliminate free radicals. Consequently, ORP may serve as an effective indicator of the prospective presence of antioxidant components [56]. This demonstrates that the low-molecular-weight protein compounds present in the AW and LP treatments had a restricted impact regarding the redox potential value of the system. The results of other research are inconclusive concerning the impact of acid whey on the oxidation-reduction potential of meat products. Wójciak et al. [65] demonstrated that including 5% acid whey in meat products reduced the redox potential of cooked pork sausages. Wójciak et al. [65] also observed comparable outcomes in fermented sausages. Whereas, Okoń et al. [25] found that adding 3% acid whey to fermented bacon resulted in an increase in oxidation-reduction potential measured after production. It is a fact that the abundance and qualitative composition of the microbiota of organic acid whey may vary [17,66], and thus it may have an impact on forming the ORP of the meat environment. The results demonstrated a significant increase in the redox potential value (*p* < 0.05) after 7 days of storage in all experimental treatments. Then, the ORP values for the C, S, and AW variants were stable, while in the case of LP, a significant decrease was observed (*p* < 0.05).

Interaction between treatment and storage time effects for TBARS value was found (*p* < 0.01) (Table 6). The highest value of the TBARS index after production was observed in the S sample (1.12 mg/kg) and the AW sample (1.08 mg/kg) (*p* < 0.05), indicating that the levels of secondary fat products formed during oxidation were the highest in these samples. A lower level of TBARS was found in the LP treatment (1.00 mg/kg) and the significantly lowest in the C sample (0.69 mg/kg), in which the lowest ORP was also observed (Table 5). Wójciak et al. [56] also observed higher levels of TBARS in roasted uncured beef with acid whey (1.57 mg/kg) than in the uncured salt sample (0.76 mg/kg) and the cured sample (0.42 mg/kg). As the cause, the authors pointed out the mechanism related to the production of H_2_ O_2_ by bacteria that are part of the acid whey microbiota. Studies have shown the effect of nitrite concentration in the product on TBARS value [67,68]. The main antioxidant activity of nitrite is the reaction of NO (formed from the reduction of NO_2_) with other radicals (hydroxyl radicals, alkoxy radicals, and peroxide radicals), which leads to the interruptions of chain reactions. An alternate potential mechanism involves the creation of a stabilised complex consisting of heme-bound iron with nitric oxide, which is then converted to a catalytically inactive form as a result of heat treatment [49]. The significantly lower value of TBARS in the LP sample after production compared to S and AW samples may be related to the involvement of *L. plantarum* S21 in antioxidant processes in raw sausage stuffing. Some environmental LAB exhibit antioxidant properties. The antioxidant components of LAB encompass a number of different elements, including bacterial exopolysaccharides (EPS), biologically active peptides, enzymatic antioxidants, and ions of manganese [15,69,70]. In *L. plantarum*, it is, among others, a pseudocatalase containing manganese (manganese-containing catalase) [71]. In the S treatment, a decrease in the TBARS value was found after 14 days of storage, which may be related to the formation of solid fat degradation complexes with other components, i.e., amino acids or sugars [52].

#### 3.2.3. Fatty Acid Profile

The content of saturated fatty acid (ƩSFA), monounsaturated fatty acid (ƩMUFA), and polyunsaturated fatty acid (ƩPUFA) in the experimental sausages is shown in Table 8. The content of individual fatty acids in the experimental sausages is shown in Supplementary Table S1.

Statistical analysis showed that treatment and time of storage affected (*p* < 0.001) the level of ƩMUFA in the sausages. An interaction between the treatment and time of storage effects for the level of ƩSFA and ƩPUFA in the products also was found. No statistically significant differences were found between the total fatty acids (ƩFAs) in the products (Table 8) (*p* > 0.05).

An interesting observation was made when comparing the fatty acid profile of sausages. It was shown that the LP and AW treatments had lower ƩSFA content and higher ƩMUFA and ƩPUFA content than the C and S treatments (*p* < 0.05). LP and AW sausages were characterised by significantly lower content of C18:0 stearic acid and C16:0 palmitic acid than C and S treatments. In turn, LP and AW sausages had significantly higher content of C18:1 n-9 c oleic acid and 18:2 n6 linoleic acid, which, as is known, is particularly susceptible to oxidation. The ƩPUFA/ƩSFA ratio of LP and AW treatments was statistically significantly higher (*p* > 0.05) than in the C and S treatments. The observed differences in the amount of fatty acids are not large quantitatively and will not have any significance in terms of the nutritional value of the products. Nevertheless, these differences indicate that acid whey and *L. plantarum* S21 or bacterial metabolites may have had a protective effect against the oxidation of unsaturated fatty acids. It cannot be ruled out that the antioxidant properties of the peptides observed in the LP and AW treatments could have played a role in this process. Another mechanism may be determined by the activity of muscle enzymes. It is known that lower muscle pH promotes lipolysis [72]. The release of MUFA may have influenced the differences in the fatty acid ratios observed after production and storage. Karwowska et al. [55] also observed a higher MUFA content (54.72%) in a sample of uncured cooked pork sausage with acid whey and mustard seeds than in a cured control sausage sample (53.99%). Whereas, Okoń et al. [25] observed a lower share of PUFA (9.55%) in uncured fermented pork belly with the addition of acid whey than in cured bacon (13.75%), with a higher share of SFA (39.00%) in bacon with whey and a lower content in the cured product (34.77%). Some studies indicate that the lipases of lactic acid bacteria are involved in the lipid metabolism of meat products [73]. Observations on the effect of LAB on the fatty acid profile of meat products have also been made in other scientific works [74,75]. In all treatments, the ƩPUFA/ƩSFA ratio decreased significantly after storage, which was related to the loss of PUFA due to hydrolysis and oxidation [25].

#### 3.2.4. Nitrosyl Pigment Content

Statistical analysis showed interaction treatment x storage time in the concentration of nitrosyl pigments in the sausages (*p* < 0.001). The highest content of nitrosyl pigments was observed in the C treatment, with a value of 64.96 ppm (*p* < 0.05). The observed changes in the content of nitrosyl pigments within the sample treatments demonstrated variability during the storage period (Table 6). A statistically significant reduction in the concentration of nitrosyl pigments during the storage period occurred in the C treatment, whereas a significantly increased concentration was observed in samples from the LP treatment (*p* < 0.05). In the S and AW treatments, no significant changes in the concentration of nitrosyl pigments during storage were observed (*p* > 0.05). Following a 14-day storage period, analysis revealed that the highest level of nitrosyl pigment concentration was present in treatments C and LP, with a lower concentration observed in AW and the lowest concentration in the S sample (*p* < 0.05) (Table 6).

The highest level of nitrosyl pigments in the C sample was associated with the addition of sodium nitrite. Nitrous acid (HNO_2_), formed from nitrites (NO_2_) added to meat, oxidises deoxymyoglobin (MbFe^2+^) to metmyoglobin (MbFe^3+^). MbFe^3+^ in meat is reduced chemically (with the contribution of –SH groups, the cysteine–cystine system or reducing substances) or biochemically (with the input of dehydrogenase or by the coenzyme NADH or FAD). In the same way, the reduction of NO_2_ to NO takes place, which then attaches to MbFe^2+^ to form MbFe^2+^NO [3]. The formation of nitric oxide can occur in the presence of bacterial enzymes [13,76], which may account for the elevated nitrosyl pigment concentration observed in the LP and AW treatments relative to the S treatment (Table 6). It has been demonstrated that certain Lactobacillus species are implicated in the transformation of MbFe^2+^ to MbFe^2+^NO in model systems and meat products [14,77]. In previous studies, this mechanism was observed in a model meat product involving *L. fermentum* S8 derived from organic acid whey [24]. However, it is still unclear how these bacteria can produce NO without adding NO_2_/NO_3_ [13]. One of the hypotheses is the mechanism of NO formation from L-arginine with the contribution of bacterial nitric oxide synthase (NOS), which can react with Mb and thus form MbFe^2+^NO [78]. Although similar activity to NOS has been reported for many bacteria, only a few NOS bacterial homologues have been determined in mammals [13,79]. Concerning the species of Lactobacillus, it is assumed that some possess genes allowing them to encode the NOS protein. Some studies indicate that L-arginine induces the expression of bacterial NOS [80], but in some bacteria, NOS cannot be effectively induced by this substrate, but by its derivatives, e.g., L-arginine methyl ester, L-arginine ethyl ester, and N-nitro-L-arginine methyl [81]. It has also been proved that some compounds commonly used in meat processing, e.g., ascorbate, maintain NOS expression in mammals [82] and may play a role in NOS expression in Lactobacillus spp. [13]. Another probable mechanism supporting the formation of MbFe^2+^NO in meat stuffing in LP and AW treatments is acid produced by bacteria and/or the acidification of meat through the addition of acid whey determining an environment that promotes the reduction of NO_3_/NO_2_ (potential contaminations brought in with spices, meat, and water) to NO [52].

The observed increase in the amount of MbFe^2+^NO (*p* < 0.05) in the LP treatment during the entire storage period may indicate the involvement of LAB or their metabolites in the conversion of undenatured myoglobin (Mb) to nitrosyl derivatives. It is established that undenatured Mb may be present in meat products subjected to thermal processing and transform into other derived forms during cold storage [83]. On the other hand, the significant decrease in nitrosyl pigments in the C sausage during storage is a known mechanism caused by nitrosomiochromogen oxidation [84]. It is worthy of note that this relation was not confirmed in the AW sample, the amount of MbFe^2+^NO during storage was at a similar level, which may indicate a protective antioxidant effect of whey proteins. In other studies, the concentration of MbFe^2+^NO in uncured heat-treated meat ranged from 0.42 ppm to 13.39 [54,68,85,86]. In cooked meat products made from cured meat with sodium nitrite at 100 mg/kg, MbFe^2+^NO was determined at a level from 34.03 ppm to 43.0 ppm [87,88]. Whereas, Szymański et al. [24] determined MbFe^2+^NO in the amount of 16.26 ppm in a cooked canned model meat product made from non-cured pork meat and cultured with *L. fermentum* S8 (~10^7^ CFU/g) isolated from acid whey.

#### 3.2.5. Post-Production and Storage Colour Analysis

The colour of meat products is critical for the consumer’s purchasing decision. This study has shown interaction between treatment and storage time for redness (a*) and hue angle (h°) parameters of sausages (*p* < 0.001) (Table 9).

After production and storage, the LP and AW sausages had a higher value of a* parameter in comparison to the S sample. A similar relationship regarding the effect of acid whey on the redness of uncured meat product was demonstrated by other authors [22,23]. In addition, it has been shown that the LP sample was characterised by a significantly lower h° value than uncured S. The highest values of a* and the lowest values of h° were obtained in the C sample (*p* < 0.05) (Table 9). The treatment and storage time affected chroma (C*) (*p* < 0.001). No interactions treatment x storage time were found (*p* > 0.05). The mean C* values were highest for the C treatment, lower values were obtained in the LP and AW samples, and the lowest in the S treatment. All sausages showed a significant increase in C* values during the first 7 days of storage, and thereafter the C* parameter values were stable. No interaction between treatment and storage time for yellowness (b*) was demonstrated (*p* > 0.05). However, a significant effect of the treatment and storage time on the b* value was shown (*p* < 0.001). Generally, the C and LP samples had lower b* values than those observed in the S and AW treatments (*p* < 0.05) (Table 9). Wójciak et al. [23] also showed a high share of yellowness in uncured cooked sausage with acid whey compared to the control cured treatment.

The significant effect of treatment and storage time on the brightness (L*) was found (Table 9). The S sausages were characterised by the highest brightness (L*). A decrease in the average L* value was also observed in all of the sausage treatments after 14 days of storage. In our study, only treatment had a significant effect on ΔE* parameter (*p* < 0.001). A significantly lower value of the ΔE* value was found in the LP sausages than in the S and AW sausages (*p* < 0.05). The results of the instrumental colour analysis (value of a* and h° parameters) correlate with the concentration of nitrosyl pigments in the experimental sausages, which suggests that the amount of MbFe^2+^NO produced in the LP and AW treatments is the key factor in colour formation. The presented research showed that the colour of the LP sausage was more similar to that of cured products than the S sample and AW sample (ΔE* comparison). Moreover, the colour of the LP sausages was stable during storage (Table 9).

#### 3.2.6. Sensory Quality

**Figure 2:**
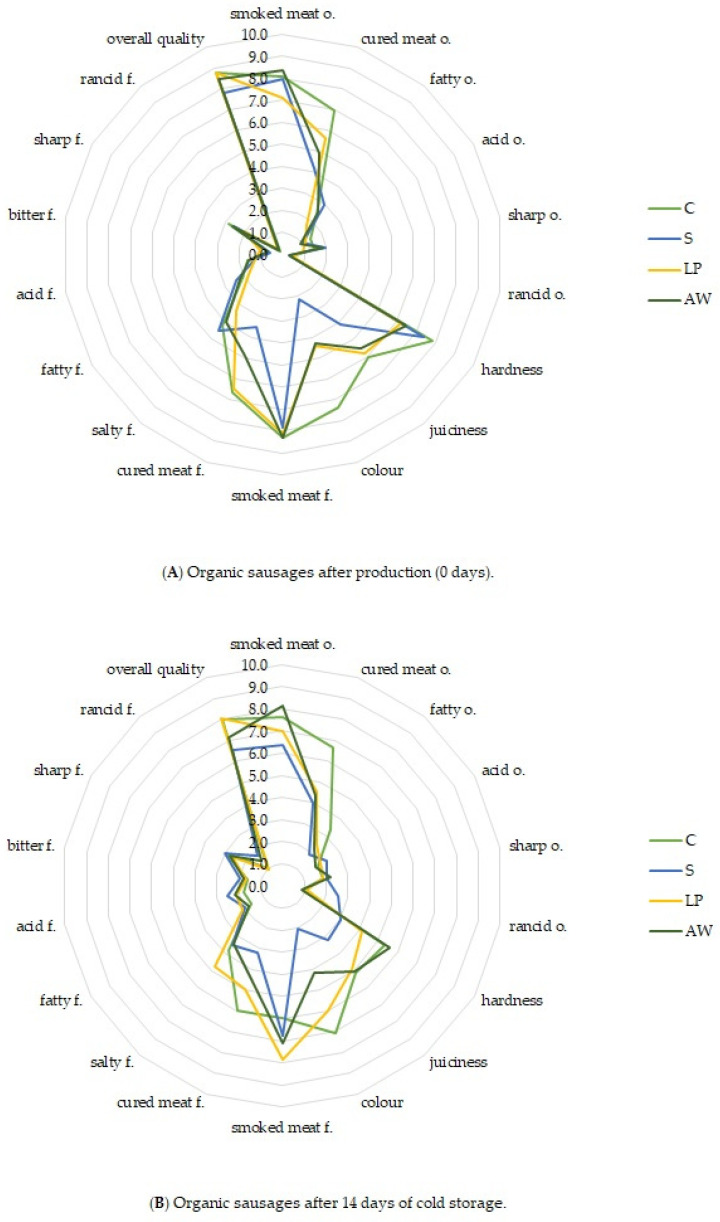
Sensory profile of experimental organic sausages after production (**A**) and after 14 days of cold storage (**B**); n = 18; and C—control cured treatment, S—salted treatment, LP—treatment with salt and *Lactiplantibacillus plantarum* S21 at about 10^7^ CFU/g, and AW—treatment with salt and acid whey.

The results of the sensory analysis of the organic sausages after production and storage conducted by the QDP method are shown in Figure 2. The statistical results of the two-way ANOVA of sensory data are summarised in Supplementary Table S2.

Statistical analysis showed a treatment × storage time interaction in the intensity of smoked meat odour, smoked meat, and rancid odour and hardness. Statistically significant effect of treatment was shown on the intensity of cured meat odour, cured meat flavour, juiciness, and colour of products. In addition, the time of storage affected intensity of acid and sharp odour, bitter and rancid flavour, juiciness, colour, and overall quality of products.

All sausages (C, S, LP, AW) tested after production (time 0) were characterised by high intensity of the odour of smoked meat (7.1–8.4 c.u.). It was reported that the control treatment (C) exhibited the highest intensity of cured meat odour, with lower scores for this parameter observed in LP and AW sausages and the lowest in the S treatment. Nevertheless, statistical significance (*p* < 0.05) in the intensity of cured meat odour was observed solely between the C and S treatments. The sausages had a similar flavour profile with a dominant hint of smoked meat (7.8–8.3 c.u.). What is important, in the LP treatment with *L. plantarum* S21, no extraneous sensory hints were found, and the intensity of such features as acid flavour, bitter flavour, and sharp flavour was at a low level, which was similar in all of the experimental treatments. Most panellists did not identify any rancid flavour in the samples they evaluated. The average scores of the flavour intensity of cured meat were higher in samples C (6.6 c.u.) and LP (6.5 c.u.) than in sample S (3.5 c.u.) (*p* < 0.05). No differences between S and AW treatments were found (*p* > 0.05).

The differences in the hardness of the evaluated products were observed. The toughest was the C sample (8.1 c.u.) and S sample (7.4 c.u.) while the LP and AW samples were significantly less tough (6.2 c.u. and 6.5 c.u., respectively) (*p* < 0.05). This observation may be related to the higher acidification of meat stuffing with *L. plantarum* S21 and acid whey, which was reported in the samples after the production (Table 6). The acidification of the meat may have had the effect of weakening structural proteins and causing the meat to become tender [89]. The tested products differed significantly in terms of the colour of the meat on the cross-section (*p* < 0.05). The cured sausage had a pink colour (7.4 c.u.), specific for MbFe^2+^NO found in cured meat products [32]. LP and AW sausages were also pink, but with a lower intensity (4.4 c.u. and 4.3 c.u., respectively). The colour of the uncured sausage was the most similar to the grey-brown colour, which was related to the low level of MbFe^2+^NO (Table 6), and received the lowest scores in the assessment of this feature (2.2 c.u.) (Figure 2). Despite the differences in the sensory profile, all the tested sausages were overall of high quality (7.8–8.8 c.u.).

After 14 days of storage, the sensory profile of the tested products changed. In general, in all samples, the intensity of the smoked odour and flavour decreased, while the intensity of the acid odour, acid flavour, and bitter flavour increased. However, these changes were not statistically significant in all samples. Statistically significant changes in rancid odour were demonstrated for the S and AW treatments. The intensity of rancid flavour increased in all tested samples (*p* < 0.05). After storage, the highest intensity of rancid flavour (1.8 c.u.) was noted in the S treatment. The remaining variants did not differ significantly (1.0–1.5 c.u.). In all treatments, a decrease in sausage hardness was observed. The S sausages were the softest, which was probably related to the greatest acidification during storage. After storage, an increase in the intensity of the pink colour was observed in the LP treatment, which was related to the increase in the amount of MbFe^2+^NO in the product. The most intense pink colour was characteristic of the C treatment (7.1 c.u.). LP and AW treatments were also pink, but with lower intensities (6.0 c.u. and 4.2 c.u., respectively) (*p* < 0.05). The colour of the uncured sausage was the most similar to the grey-brown colour. After 14 days of storage, the overall quality of all the products decreased (*p* < 0.05). This was probably related to the increased intensity of sensory characteristics such as acid, bitter, and rancid. Nevertheless, the overall quality of the products remained highly rated (6.9–8.1 c.u.). The highest overall quality was achieved by treatments C and LP (*p* < 0.05).

#### 3.2.7. Microbiological Analysis

In addition to playing a pivotal role in inhibiting the growth and proliferation of the botulinum toxin produced by Clostridium botulinum, nitrites have been observed to have an inhibiting effect on other bacterial species. Nitrites participate in the synthesis of reactive forms of nitric oxide (ONOO-/ONOOH) in meat, which have the potential to damage bacterial cells [4]. Therefore, it is very important to assess the shelf life and the microbial quality of uncured meat products. All of the tested products were free of *L. monocytogenes*, *Staphylococcus aureus*, and *Salmonella* spp. A total viable count (TVC) and lactic acid bacteria enumeration of all the sausages produced demonstrated a reading of less than 2.0 log CFU/g for both variables after production and one week of storage. The statistical analysis revealed a significant difference (*p* < 0.05) in TVC between the treatments after 14 days of storage (Table 10). A significantly lower TVC was found in the C and LP treatments than in the S and AW treatments. TVC in treatment C was probably related to the effect of added NO_2_ on bacteria. On the other hand, the low TVC observed in the LP treatment indicates an inhibitory effect of *L. plantarum* S21 or its metabolites/postbiotics on other bacteria. This may be due to the effect of competitive interactions between microorganisms. After 14 days of storage, the lactic acid bacteria were identified at a concentration of 2.42 log CFU/g in the LP treatment (Table 10).

The research on the evaluation of the antimicrobial properties of lactic acid bacteria isolated from organic acid whey suggests that the *L. plantarum* S21 strain has the ability to produce bacteriocins or bacteriocin-like substances [17]. In our research, the addition of organic acid whey did not reduce the TVC. Similar results were observed by other researchers in cooked pork sausage with 5% organic acid whey added. The TVC of the control sausage (7.17 log CFU/g) was comparable to that of the experimental sausage with acid whey (8.58 log CFU/g) after 30 days of refrigerator storage [23]. No Clostridium spp. was found in the tested products, which indicates the quality of the raw material used for sausage production and confirms no contamination with these bacteria. However, the risks that may arise when producing meat products without adding nitrites in terms of *Clostridium botulinum* contamination should not be underestimated. The hygiene at animal slaughterhouses and meat-cutting plants in developed countries is currently at a high level, however, there is always the risk of raw material contamination with *Clostridium botulinum* bacteria. Therefore, to ensure the health safety of uncured meat products, it is important to analyse the risk at the level of the production plant and implement additional anti-botulinum barriers (reducing pH, lowering aw, increasing sodium chloride concentration, proper management of the cold supply chain, no use of packaging in anaerobic conditions, and shortening storage time) in production and distribution [90,91].

## 4. Conclusions

This study demonstrates that the application of *Lactiplantibacillus plantarum* S21 form acid whey in the production of heat-treated, nitrite-free, organic sausages can effectively contribute to the formation of desirable sensory attributes and microbiological stability, making it a promising alternative to traditional nitrite curing. The results confirm that *L. plantarum* S21 enhances the pink colour formation, achieving levels comparable to nitrite-treated control samples. Moreover, sausages inoculated with this strain exhibited good microbiological quality, stable colour, and acceptable sensory characteristics over the storage period, further supporting the feasibility of this approach. However, it is important to acknowledge that the two microbiological and physicochemical properties of the sausages remained stable. Further research is needed to evaluate the long-term effects of storage and potential variations in sensory characteristics over extended periods. Our findings also indicated that using selected pure cultures of lactic acid bacteria isolated from organic acid whey in nitrite-free meat processing can be more effective than using acid whey as an additive. However, in contrast to previous reports on the antioxidant potential of lactic acid bacteria, our findings do not clearly indicate the antioxidant effect of *L. plantarum* S21. We observed that *L. plantarum* S21 or bacterial metabolites may have had a protective effect against the oxidation of unsaturated fatty acids; on the other hand, we did not observe TBARS values as low as those in the cured sausages. This is particularly relevant in the context of nitrite-free meat processing, where oxidation control remains a challenge.

From a practical standpoint, the suggested technology may be a promising solution to produce high-quality organic meat products without the addition of sodium nitrite assuming the implementation of additional anti-botulinum barriers. Additionally, future studies should explore the scalability of this approach in diverse meat matrices and under different production conditions to validate its industrial applicability. Overall, this research contributes valuable insights into the development of organic meat products, aligning with consumer demands for healthier and more natural food alternatives.

## Figures and Tables

**Table 1:** Strains of lactic acid bacteria used in the experiment.

Strain	GenBank Accession	Source of Isolation	Reference
*Lactiplantibacillus plantarum* S1	KY363517	Raw, non-pasteurised, organic acid whey	[17]
*Limosilactobacillus fermentum* S4	KY363556		
*Limosilactobacillus fermentum* S7	KY363569		
*Limosilactobacillus fermentum* S8	KY363557		
*Limosilactobacillus fermentum* S10	KY363646		
*Lactiplantibacillus plantarum* S11	KY363559		
*Lactiplantibacillus plantarum* S16	KY363650		
*Lactiplantibacillus plantarum* S17	KY363648		
*Limosilactobacillus fermentum* S18	KY363554		
*Lactiplantibacillus plantarum* S19	KY363652		
*Lactiplantibacillus plantarum* S21	KY363566		

**Table 2:** Recipe of model meat products.

Component	Treatment		
	C1	C2	S1, S4, S7, S8, S10, S11, S16, S17, S18, S19, S21
Pork ham muscle *M. semimembranosus* (kg)	5	5	5
NaCl (kg)	0.1	0.1	0.1
NaNO_2_ (g)	0.5	-	-
Bacterial biomass of strain (Table 1) suspended in 0.85% NaCl solution (g)	-	-	50
0.85% NaCl solution (g)	50	50	-
Glucose (g)	50	50	50
Demineralised water (kg)	0.25	0.25	0.25

C1—control treatment with salt and NaNO_2_ at 100 mg/kg; C2—treatment with salt; and S1, S4, S7, S8, S10, S11, S16, S17, S18, S19, S21—treatments with salt and strains of lactic acid bacteria (Table 1) at about 10^7^ CFU/g.

**Table 3:** Recipe of heat-treated organic sausage.

Component (kg)	Treatment			
	C	S	LP	AW
Pork ham muscles (*M. semimembranosus*, *M. semitendinosus*, and *M. biceps femoris*)	80	80	80	80
Fatty meat from pork ham—trimmings	20	20	20	20
NaCl	-	1.592	1.592	1.592
Nitrite curing mixture (99.5% NaCl, 0.5% NaNO_2_)	1.6	-	-	-
Bacterial biomass *L. plantarum* S21 suspended in 0.85% NaCl solution	-	-	1	-
0.85% NaCl solution	1	1	-	1
Acid whey	-	-	-	5
Water/ice	7	7	7	2
Glucose	1	1	1	1
Black pepper	0.43	0.43	0.43	0.43
Coriander	0.11	0.11	0.11	0.11
Fresh garlic	0.21	0.21	0.21	0.21

C—control cured treatment, S—salted treatment, LP—treatment with salt and *Lactiplantibacillus plantarum* S21 at about 10^7^ CFU/g, and AW—treatment with salt and acid whey.

**Table 4:** Colour parameters (L*, a*, b*, h*, C*) and pH values in the experimental pork cooked batters (means ± SE).

Treatment	L*	a*	b*	h*	C*	pH
C1	66.1 ± 0.1 ^a^	12.3 ± 0.4 ^g^	1.7 ± 0.4 ^a^	8.1 ± 2.1 ^a^	12.5 ± 0.6 ^f^	6.34 ± 0.02 ^d^
C2	69.0 ± 0.6 ^f^	8.1 ± 0.1 ^a^	4.6 ± 0.1 ^g^	29.6 ± 1.0 ^f^	9.4 ± 0.2 ^bc^	6.35 ± 0.02 ^d^
S1	67.4 ± 0.2 ^b^	8.6 ± 0.1 ^cd^	4.0 ± 0.1 ^f^	24.6 ± 0.7 ^e^	9.5 ± 0.1 ^bcd^	6.13 ± 0.03 ^bc^
S4	67.7 ± 0.8 ^bc^	8.4 ± 0.1 ^abc^	4.0 ± 0.1 ^f^	25.2 ± 1.0 ^e^	9.3 ± 0.2 ^ab^	6.10 ± 0.01 ^abc^
S7	68.2 ± 0.1 ^cde^	8.9 ± 0.1 ^de^	3.7 ± 0.1 ^cdef^	22.6 ± 0.8 ^cd^	9.6 ± 0.1 ^cd^	6.11 ± 0.01 ^abc^
S8	66.3 ± 0.5 ^a^	9.0 ± 0.3 ^e^	3.7 ± 0.2 ^def^	22.7 ± 1.1 ^cd^	9.7 ± 0.6 ^d^	6.12 ± 0.04 ^abc^
S10	68.6 ± 0.5 ^ef^	8.6 ± 0.1 ^c^	3.4 ± 0.1 ^c^	21.8 ± 1.8 ^c^	9.2 ± 0.2 ^ab^	6.10 ± 0.02 ^abc^
S11	67.8 ± 0.1 ^bcd^	8.7 ± 0.2 ^cde^	3.4 ± 0.1 ^bcd^	21.8 ± 1.1 ^c^	9.3 ± 0.2 ^ab^	6.14 ± 0.03 ^c^
S16	67.8 ± 0.3 ^bcd^	8.5 ± 0.1 ^bc^	3.5 ± 0.2 ^bcde^	22.3 ± 1.1 ^cd^	9.2 ± 0.1 ^ab^	6.14 ± 0.03 ^c^
S17	68.0 ± 0.2 ^bcde^	8.7 ± 0.2 ^cde^	3.8 ± 0.1 ^f^	23.9 ± 1.2 ^de^	9.5 ± 0.2 ^bcd^	6.11 ± 0.03 ^abc^
S18	68.0 ± 0.2 ^bcde^	8.2 ± 0.1 ^ab^	3.8 ± 0.1 ^ef^	24.7 ± 0.6 ^e^	9.1 ± 0.1 ^a^	6.10 ± 0.01 ^abc^
S19	68.4 ± 0.3 ^def^	8.6 ± 0.1 ^cd^	3.4 ± 0.1 ^bcd^	21.9 ± 0.3 ^c^	9.3 ± 0.2 ^ab^	6.08 ± 0.02 ^a^
S21	68.1 ± 0.1 ^cde^	9.4 ± 0.1 ^f^	3.3 ± 0.1 ^b^	19.6 ± 1.1 ^b^	10.0 ± 0.2 ^e^	6.09 ± 0.02 ^ab^

^(a–f)^ Means with the different letters between the treatments differ significantly (*p* < 0.05); n = 3; SE: standard error; C1—control treatment with salt and NaNO_2_ at 100 mg/kg; C2—treatment with salt; and S1, S4, S7, S8, S10, S11, S16, S17, S18, S19, S21—treatments with salt and strains of lactic acid bacteria (Table 1) at about 10^7^ CFU/g.

**Table 5:** Sensory analysis of the experimental pork cooked batters (mean panellist ratings ± SE).

Treatment	Discriminants			
	Overall Appearance	Consistency	Odour	Flavour
C2	2.1 ± 0.3 ^ab^	6.9 ± 0.5 ^cd^	3.1 ± 0.5 ^a^	3.8 ± 0.5 ^a^
S1	1.9 ± 0.3 ^a^	7.3 ± 0.4 ^d^	4.0 ± 0.5 ^ab^	4.3 ± 0.5 ^a^
S4	3.2 ± 0.4 ^bc^	6.2 ± 0.4 ^bcd^	3.9 ± 0.4 ^ab^	4.6 ± 0.5 ^ab^
S7	3.9 ± 0.4 ^cd^	7.1 ± 0.5 ^cd^	6.3 ± 0.6 ^d^	6.3 ± 0.4 ^c^
S8	4.1 ± 0.4 ^d^	7.3 ± 0.4 ^d^	3.9 ± 0.4 ^ab^	4.9 ± 0.4 ^ab^
S10	2.6 ± 0.3 ^ab^	5.3 ± 0.6 ^ab^	4.7 ± 0.3 ^bc^	4.2 ± 0.5 ^a^
S11	2.3 ± 0.3 ^ab^	6.0 ± 0.6 ^abc^	4.3 ± 0.4 ^abc^	4.4 ± 0.5 ^a^
S16	2.6 ± 0.5 ^ab^	6.3 ± 0.4 ^bcd^	4.6 ± 0.5 ^bc^	5.6 ± 0.3 ^bc^
S17	3.9 ± 0.4 ^cd^	7.3 ± 0.5 ^d^	5.4 ± 0.4 ^cd^	6.9 ± 0.4 ^c^
S18	2.1 ± 0.3 ^ab^	6.7 ± 0.5 ^cd^	4.0 ± 0.4 ^ab^	4.5 ± 0.5 ^ab^
S19	2.6 ± 0.3 ^ab^	4.8 ± 0.6 ^a^	4.0 ± 0.3 ^ab^	4.4 ± 0.4 ^ab^
S21	5.4 ± 0.4 ^e^	7.1 ± 0.5 ^cd^	5.4 ± 0.5 ^cd^	6.3 ± 0.5 ^c^

^a–e^ Means with the different letters between the treatments differ significantly (*p* < 0.05); n = 18; SE: standard error; C2—treatment with salt; and S1, S4, S7, S8, S10, S11, S16, S17, S18, S19, S21—treatments with salt and strains of lactic acid bacteria (Table 1) at about 10^7^ CFU/g.

**Table 6:** The pH, ORP, TBARS, and MbFe^2+^NO concentration in the experimental organic sausages (means ± SE).

Parameter	Treatment	0th Day	7th Day	14th Day	*p*_t_	*p*_s_	*p*_t × s_
pH	C	6.16 ± 0.04 ^e^	5.84 ± 0.09 ^ab^	6.12 ± 0.02 ^de^	*	**	*
	S	6.07 ± 0.02 ^cde^	5.75 ± 0.11 ^a^	5.87 ± 0.04 ^ab^			
	LP	5.78 ± 0.02 ^ab^	5.87 ± 0.16 ^ab^	5.97 ± 0.03 ^bcd^			
	AW	5.87 ± 0.09 ^ab^	5.84 ± 0.08 ^ab^	5.90 ± 0.07 ^abc^			
OPR (mv)	C	317.6 ± 7.4 ^a^	366.7 ± 1.6 ^cd^	356.3 ± 8.6 ^c^	*	***	*
	S	331.1 ± 6.4 ^b^	374.9 ± 1.0 ^de^	360.9 ± 3.1 ^cd^			
	LP	332.6 ± 1.3 ^b^	381.5 ± 2.8 ^e^	364.2 ± 6.9 ^cd^			
	AW	336.8 ± 4.5 ^b^	370.1 ± 6.0 ^cde^	361.4 ± 6.5 ^cd^			
TBARS	C	0.69 ± 0.03 ^a^	0.81 ± 0.05 ^b^	0.74 ± 0.04 ^ab^	***	NS	**
(mg MDA/kg)	S	1.12 ± 0.02 ^f^	0.96 ± 0.01 ^cd^	0.93 ± 0.03 ^c^			
	LP	1.00 ± 0.02 ^cd^	1.10 ± 0.03 ^f^	1.04 ± 0.02 ^def^			
	AW	1.08 ± 0.01 ^ef^	1.06 ± 0.04 ^ef^	1.05 ± 0.02 ^def^			
MbFe^2+^NO	C	65.96 ± 1.37 ^i^	42.63 ± 2.13 ^h^	31.90 ± 1.17 ^g^	***	***	***
(ppm)	S	10.37 ± 1.06 ^a^	12.57 ± 0.69 ^ab^	14.84 ± 2.12 ^b^			
	LP	20.49 ± 1.70 ^c^	25.86 ± 0.55 ^ef^	28.52 ± 1.02 ^fg^			
	AW	21.07 ± 0.44 ^c^	24.84 ± 0.84 ^de^	21.46 ± 3.19 ^cd^			

^(a–i)^ Means with the different letters differ significantly (*p* < 0.05); n = 3; SE: standard error; *p*: significance of effects; treatment (t); time of storage (s); treatment × time of storage interaction (t × s); NS—not significant; * *p* < 0.05; ** *p* < 0.01; *** *p* < 0.001; and C—control cured treatment, S—salted treatment, LP—treatment with salt and *Lactiplantibacillus plantarum* S21 at about 10^7^ CFU/g, and AW—treatment with salt and acid whey.

**Table 7:** ABTS•+ parameter of experimental organic sausages tested after production (means ± SE).

Parameter/Treatment	C	S	LP	AW
ABTS•+ (mM Trolox/ mg peptides)	38.15 ± 0.11 ^b^	32.28 ± 0.86 ^a^	42.73 ± 0.71 ^c^	47.28 ± 0.71 ^d^

Means with different superscript letters differ significantly (*p* < 0.05); n = 3; SE: standard error; and C—control cured treatment, S—salted treatment, LP—treatment with salt and *Lactiplantibacillus plantarum* S21 at about 10^7^ CFU/g, and AW—treatment with salt and acid whey.

**Table 8:** Fatty acid content (mg/100 g) of experimental organic sausages (means ± SE).

Storage Time	0th Day				14th Day							
Treatment	C	S	LP	AW	C	S	LP	AW	SEM	*p*_t_	*p*_s_	*p*_t × s_
ƩSFA	5716.8 ± 24.8 ^cde^	5702.1 ± 21.9 ^bcd^	5589.4 ± 21.0 ^a^	5615.1 ± 20.0 ^a^	5728.2 ± 38.9 ^de^	5755.2 ± 20.7 ^e^	5624.4 ± 22.9 ^abc^	5600.3 ± 41.0 ^ab^		***	***	**
ƩMUFA	7056.5 ± 32.8	7124.1 ± 27.6	7308.1 ± 28.4	7316.8 ± 26.4	7184.7 ± 25.5	7198.8 ± 24.9	7303.0 ± 25.8	7165.3 ± 19.3		***	***	NS
Means *p*_t_	7140.4 ^o^	7165.2 ^o^	7305.5 ^p^	7224.0 ^p^					17.8			
ƩPUFA	1331.1 ± 11.7 ^de^	1313.7 ± 15.8 ^d^	1451.1 ± 12.3 ^ef^	1444.7 ± 8.3 ^f^	1183.6 ± 18.2 ^a^	1192.6 ± 11 ^a^	1288.1 ± 7.9 ^c^	1225.4 ± 17.3 ^b^		***	***	***
ƩFAs	14,104.4 ± 70.0	14,139.9 ± 48.6	14,348.6 ± 50.8	14,376.6 ± 48.6	14,096.5 ± 50.9	14,146.6 ± 70.9	14,215.5 ± 42.0	13,991.0 ± 53.1		NS	NS	NS
ƩPUFA/ƩSFA	0.23 ± 0.00 ^d^	0.23 ± 0.00 ^d^	0.26 ± 0.00 ^e^	0.26 ± 0.00 ^e^	0.20 ± 0.00 ^a^	0.21 ± 0.00 ^a^	0.23 ± 0.00 ^c^	0.25 ± 0.00 ^b^		***	**	***

^a–f^ Means with different letters differ significantly (*p* < 0.05). ^o–p^ Means with different letters differ significantly (*p* < 0.05) within the treatment. n = 3; SEM: standard error of means; SFA: saturated fatty acid; MUFA: monounsaturated fatty acid; PUFA: polyunsaturated fatty acid; FA: total fatty acid; *p*: significance of effects; treatment (t); time of storage (s); treatment × time of storage interaction (t × s); NS—not significant; ** *p* < 0.01; *** *p* < 0.001; and C—control cured treatment, S—salted treatment, LP—treatment with salt and *Lactiplantibacillus plantarum* S21 at about 10^7^ CFU/g, and AW—treatment with salt and acid whey.

**Table 9:** Effect of treatment and storage time on the L*, a *, b*, h°, C*, and ΔE* values of experimental organic sausages during chilled storage (means ± SE).

Parameter	Treatment	0 Days	7 Days	14 Days	Means	SEM	*p*_t_	*p*_s_	*p*_t × s_
L*	C	64.3 ± 1.5	64.7 ± 0.7	61.0 ± 1.3	63.3 ^x^		**	***	NS
	S	65.8 ± 1.3	65.1 ± 1.0	63.6 ± 0.4	64.8 ^y^				
	LP	64.1 ± 1.3	62.6 ± 1.2	62.7 ± 0.8	63.1 ^x^				
	AW	62.8 ± 1.7	64.3 ± 0.9	61.2 ± 2.4	62.8 ^x^	0.8			
	Means	64.2 ^p^	64.2 ^p^	62.1 ^o^		0.9			
a*	C	13.3 ± 0.5 ^f^	14.3 ± 0.5 ^g^	14.2 ± 0.5 ^g^			***	***	*
	S	8.5 ± 0.5 ^a^	10.9 ± 0.4 ^bcd^	10.4 ± 0.2 ^b^					
	LP	11.1 ± 1.3 ^bcd^	12.5 ± 0.8 ^ef^	12.5 ± 0.7 ^ef^					
	AW	10.7 ± 0.4 ^bc^	11.3 ± 0.4 ^cd^	11.7 ± 0.5 ^de^					
b*	C	5.0 ± 0.4	4.4 ± 0.4	5.2 ± 0.3	4.9 ^x^		***	*	NS
	S	6.3 ± 0.5	5.9 ± 0.4	6.0 ± 0.5	6.1 ^y^				
	LP	5.5 ± 0.4	4.4 ± 0.5	5.2 ± 0.2	5.0 ^x^				
	AW	5.6 ± 0.5	6.0 ± 0.5	6.1 ± 0.1	5.9 ^y^	0.2			
	Means	5.6 ^p^	5.2 ^o^	5.6 ^p^		0.1			
hº	C	20.7 ± 3.0 ^ab^	17.2 ± 1.7 ^a^	20.2 ± 1.6 ^ab^			***	***	*
	S	36.0 ± 2.8 ^d^	28.3 ± 1.5 ^c^	29.6 ± 2.4 ^c^					
	LP	26.2 ± 2.3 ^c^	19.3 ± 2.4 ^ab^	22.5 ± 1.4 ^b^					
	AW	27.4 ± 2.9 ^c^	27.6 ± 1.6 ^c^	27.5 ± 3.0 ^c^					
C*	C	14.2 ± 0.5	15.0 ± 0.5	15.2 ± 0.5	14.8 ^z^		***	***	NS
	S	10.7 ± 0.5	12.4 ± 0.5	12.1 ± 0.2	11.7 ^x^				
	LP	12.4 ± 0.5	13.3 ± 0.8	13.6 ± 0.6	13.1 ^y^				
	AW	12.1 ± 0.2	12.8 ± 0.4	13.2 ± 0.2	12.7 ^y^	0.2			
	Means	12.3 ^o^	13.4 ^p^	13.5 ^p^		0.2			
∆E*	C								
	S	6.5 ± 0.9		5.3 ± 0.8	5.9 ^y^		***	NS	NS
	LP	3.9 ± 0.7		2.9 ± 0.9	3.4 ^x^				
	AW	4.9 ± 0.9		5.4 ± 0.9	5.2 ^y^	0.4			

^a–g^ Means with different letters differ significantly (*p* < 0.05). ^o–p^ Means with different letters differ significantly (*p* < 0.05) within the treatment. ^x–z^ Means with different letters differ significantly (*p* < 0.05) within the storage time; n = 3; SEM: standard error of means; *p*: significance of effects; treatment (t); time of storage (s); treatment × time of storage interaction (t × s); NS—not significant; * *p* < 0.05; ** *p* < 0.01; *** *p* < 0.001; and C—control cured treatment, S—salted treatment; LP—treatment with salt and *Lactiplantibacillus plantarum* S21 at about 10^7^ CFU/g, and AW—treatment with salt and acid whey.

**Table 10:** Microbiological quality of the experimental organic sausages (means ± SE).

Microorganisms [log CFU/g]	Treatment	Storage Time [Days]		
		0	7	14
Total viable counts	C	<2.00	<2.00	2.88 ^a^ ± 0.25
	S	<2.00	<2.00	3.80 ^b^ ± 0.28
	LP	<2.00	<2.00	2.81 ^a^ ± 0.28
	AW	<2.00	<2.00	4.10 ^b^ ± 0.12
Lactic acid bacteria	C	<2.00	<2.00	<2.00 ^a^
	S	<2.00	<2.00	<2.00 ^a^
	LP	<2.00	<2.00	2.42 ^b^ ± 0.22
	AW	<2.00	<2.00	<2.00 ^a^
*Staphylococcus aureus*	C	N.D.	N.D.	N.D.
	S	N.D.	N.D.	N.D.
	LP	N.D.	N.D.	N.D.
	AW	N.D.	N.D.	N.D.
*Listeria monocytogenes* presence in 25 g	C	N.D.	N.D.	N.D.
	S	N.D.	N.D.	N.D.
	LP	N.D.	N.D.	N.D.
	AW	N.D.	N.D.	N.D.
*Salmonella* spp. presence in 25 g	C	N.D.	N.D.	N.D.
	S	N.D.	N.D.	N.D.
	LP	N.D.	N.D.	N.D.
	AW	N.D.	N.D.	N.D.
*Clostridium* spp.	C	N.D.	N.D.	N.D.
	S	N.D.	N.D.	N.D.
	LP	N.D.	N.D.	N.D.
	AW	N.D.	N.D.	N.D.

^(a–b)^ Means followed by the different case letters between the treatment are significantly different (*p* < 0.05); n = 3; SE: standard error; N.D.—not detected; and C—control cured treatment, S—salted treatment, LP—treatment with salt and *Lactiplantibacillus plantarum* S21 at about 10^7^ CFU/g, and AW—treatment with salt and acid whey.

## Data Availability

The original data obtained in the study are included in the article and Supplementary Material. Further inquiries can be directed to the corresponding author.

## Supplementary Materials

The following supporting information can be downloaded at https://www.mdpi.com/article/10.3390/foods14061028/s1: Table S1: The content of individual fatty acids (mg/100 g) in the experimental sausages (means ± SE); and Table S2: Sensory discriminants of the experimental organic sausages after production (0 days) and 14 days of cold storage (mean panellist ratings ± SE).
