# Supplementary material for: Use of Selected Environmental Lactic Acid Bacteria During Industrial Production of Heat-Treated Nitrite-Free Organic Sausage
Source: Foods. 2025 Mar 18;14(6):1028. doi: 10.3390/foods14061028 (PMC11941634; doi:10.3390/foods14061028)
Supplement: Supplementary file 1 [file foods-14-01028-s001.zip › foods-3493635-supplementary.pdf]

**Supplementary**

Table S1. The content of individual fatty acids (mg/100 g) in the experimental sausages (means ± SE).

| Storage time | 0th day | | | | 14th day | | | | | | | |
|---|---|---|---|---|---|---|---|---|---|---|---|---|
| Treatment | C | S | LP | AW | C | S | LP | AW | SEM | $p_t$ | $p_s$ | $p_{t\times s}$ |
| C10:0 | 14.2±0.1 | 14.2±0.1 | 14.2±0.1 | 14.2±0.1 | 14.1±0.2 | 14.2±0.1 | 14.2±0.1 | 14.2±0.1 | | N.S. | N.S. | N.S. |
| C12:0 | 14.2±0.1 | 14.2±0.1 | 14.2±0.1 | 14.2±0.1 | 14.1±0.2 | 14.2±0.1 | 14.2±0.1 | 14.2±0.1 | | N.S. | N.S. | N.S. |
| C14:0 | 176.9±7.7 | 169.8±1.1 | 169.8±1 | 176.9±6.5 | 183.3±2.3 | 184.0±1.1 | 184.0±1.1 | 184.0±1.1 | | N.S. | *** | N.S. |
| Means $p_s$ | | | 171.6$^{x}$ | | 183.8$^{y}$ | | | | 1.9 | | | |
| C16:0 | 3555.2±14.1 | 3534±12.0 | 3509.2±13.9 | 3510.1±14.4 | 3567.2±21.4 | 3580.0±12.9 | 3530.5±15.5 | 3537.6±18.8 | | ** | *** | N.S. |
| Means $p_t$ | 3561.2$^{p}$ | 3557.0$^{p}$ | 3519.8$^{o}$ | 3523.4$^{o}$ | | | | | 10.8 | | | |
| Means $p_s$ | 3526.9$^{x}$ | | | | 3553.8$^{y}$ | | | | 7.7 | | | |
| C17:0 | 31.8±3.7$^{ab}$ | 52.5±0.3$^{c}$ | 28.3±0.2$^{a}$ | 35.4±7.2$^{ab}$ | 31.8±6.3$^{ab}$ | 52.5±0.3$^{c}$ | 28.3±0.2$^{a}$ | 35.4±7.2$^{ab}$ | | *** | *** | *** |
| C18:0 | 1910.3±6.9 | 1903.2±9.8 | 1839.5±6.6 | 1850.1±6.4 | 1903.5±13.8 | 1896.1±6.8 | 1839.5±6.6 | 1800.7±18.5 | | *** | N.S. | N.S. |
| Means $p_t$ | 1906.9$^{p}$ | 1900.0$^{p}$ | 1820.1$^{o}$ | 1839.5$^{o}$ | | | | | 7.2 | | | |
| C20:0 | 14.2±0.1 | 14.2±0.1 | 14.2±0.1 | 14.2±0.1 | 14.2±0.1 | 14.2±0.1 | 14.2±0.1 | 14.2±0.1 | | N.S. | N.S | N.S. |
| C16:1 | 438.7±2.7 | 462.8±2.8 | 438.7±2.7 | 462.8±2.8 | 442.2±7.5 | 462.8±2.8 | 462.8±2.8 | 462.8±2.8 | | N.S. | N.S | N.S. |
| C17:1 | 28.3±0.2 | 35.4±7 | 35.4±7.2 | 31.8±5.9 | 39±6.3 | 42.5±0.3 | 42.5±0.3 | 42.5±0.3 | | N.S. | N.S. | N.S. |
| C18:1trans | 17.7±6.2 | 28.3±0.2 | 28.3±0.2 | 17.7±6.2 | 28.3±0.2 | 28.3±0.2 | 28.3±0.2 | 28.3±0.2 | | N.S. | N.S. | N.S. |
| C18:1n-9c | 5935.9±14.4 | 5946.6±22.9 | 6140.6±22.4 | 6150.0±24.0 | 5996±16.0 | 5992.5±19.4 | 6104.3±24.8 | 5950.1±23.3 | | *** | N.S. | N.S. |

| | | | | | | | | | | | | |
|---|---|---|---|---|---|---|---|---|---|---|---|---|
| Means $p_t$ | 5966.0$^{o}$ | 5969.5$^{o}$ | 6122.5$^{p}$ | 6050.5$^{p}$ | | | | | 14.8 | | | |
| C18:1n-11c | 523.6±1.9 | 534.2±3.5 | 537.7±1.9 | 537.7±1.9 | 523.6±1.9 | 527.1±4.6 | 537.7±1.9 | 554.2±3.4 | | *** | N.S. | N.S. |
| Means $p_t$ | 523.55$^{o}$ | 530.6$^{p}$ | 537.7$^{r}$ | 545.9$^{r}$ | | | | | 4.0 | | | |
| C20:1 | 113.2±0.7 | 116.8±6.4 | 127.4±0.8 | 116.8±6.4 | 127.4±0.8 | 127.4±0.8 | 127.4±0.8 | 127.4±0.8 | | N.S. | N.S. | N.S. |
| Means $p_t$ | 7140.4$^{o}$ | 7165.2$^{o}$ | 7305.5$^{p}$ | 7224.0$^{p}$ | | | | | 17.8 | | | |
| C18:2n6 | 1033.0±3.7$^{c}$ | 1002.9±8.0$^{c}$ | 1140.0±6.9$^{d}$ | 1133±6.3$^{d}$ | 922.7±5.2$^{a}$ | 923.3±5.6$^{a}$ | 990.5±6.1$^{c}$ | 957.0±14.9$^{b}$ | | *** | *** | *** |
| C18:3n6 | 14.2±0.1 | 14.1±0.1 | 14.2±0.1 | 14.2±0.1 | 14.1±0.2 | 14.2±0.1 | 14.2±0.1 | 14.1±0.1 | | N.S. | N.S. | N.S. |
| C18:3n3 | 70.8±0.4$^{b}$ | 56.5±0.5$^{a}$ | 70.8±0.4$^{b}$ | 70.8±0.4$^{b}$ | 70.5±0.9$^{b}$ | 70.8±0.4$^{b}$ | 70.8±0.4$^{b}$ | 70.6±0.6$^{b}$ | | *** | *** | *** |
| C18:2c9t11 | 14.2±0.1 | 14.1±0.1 | 14.2±0.1 | 14.2±0.1 | 14.1±0.2 | 14.2±0.3 | 14.2±0.1 | 14.1±0.1 | | N.S. | N.S. | N.S. |
| C20:2 | 42.5±0.3 | 42.4±0.4 | 42.5±0.3 | 42.5±0.3 | 42.3±0.5 | 42.5±0.3 | 42.5±0.3 | 42.4±0.4 | | N.S. | N.S. | N.S. |
| C20:3n6 | 14.2±0.1 | 14.1±0.1 | 14.2±0.1 | 14.2±0.1 | 14.1±0.2 | 14.2±0.1 | 14.2±0.1 | 14.1±0.1 | | N.S. | N.S. | N.S. |
| C20:4n6 | 102.6±6.4$^{c}$ | 127.1±1.2$^{e}$ | 113.2±0.7$^{d}$ | 113.2±0.7$^{d}$ | 63.5±7.5$^{a}$ | 70.8±0.4$^{b}$ | 99.1±0.6$^{c}$ | 70.6±0.6$^{b}$ | | *** | *** | *** |
| C20:3n3 | 14.2±0.1 | 14.1±0.1 | 14.2±0.1 | 14.2±0.1 | 14.1±0.2 | 14.2±0.1 | 14.2±0.1 | 14.1±0.1 | | N.S. | N.S. | N.S. |
| C22:4n6 | 14.2±0.1 | 14.2±0.1 | 14.2±0.1 | 14.2±0.1 | 14.1±0.2 | 14.2±0.1 | 14.2±0.1 | 14.1±0.1 | | N.S. | N.S. | N.S. |
| C22:5n3 | 14.2±0.1 | 14.1±0.1 | 14.2±0.1 | 14.2±0.1 | 14.1±0.2 | 14.2±0.1 | 14.2±0.1 | 14.1±0.1 | | N.S. | N.S. | N.S. |

$^{a\text{-}e}$ Means with different letters differ significantly ($p < 0.05$). $^{o\text{-}r}$ Means with different letters differ significantly ($p < 0.05$) within the treatment. $^{x\text{-}y}$ Means with different letters differ significantly ($p < 0.05$) within the storage time. n=3. SEM: standard error of means. *p*: significance of effects; treatment (t); time of storage (s); treatment × time of storage interaction (t×s); NS – not significant; ** $p < 0.01$; ***$p < 0.001$. C - control cured treatment, S – salted treatment, LP - treatment with a salt and *Lactiplantibacillus plantarum* S21 at about $10^7$ CFU /g, AW - treatment with a salt and acid whey.

Table S2. Sensory discriminants of the experimental organic sausages after production ( 0 days) and 14 days of cold storage (mean panellists ratings ± SE).

| Discriminants | Storage time | Treatment | | | | |
|---|---|---|---|---|---|---|
| | | C | S | LP | AW | ANOVA |
| smoked meat odour | 0 days | 8.1±0.2$^{b}$ | 8.0±0.2$^{bB}$ | 7.1±0.3$^{a}$ | 8.4±0.2$^{b}$ | T×S*** |
| | 14 days | 7.6±0.2$^{b}$ | 6.4±0.4$^{aA}$ | 7.0±0.2$^{b}$ | 8.1±0.2$^{b}$ | |
| cured meat odour | 0 days | 6.9±0.6$^{b}$ | 4.2±0.6$^{a}$ | 5.6±0.5$^{ab}$ | 4.9±0.7$^{a}$ | T*** |
| | 14 days | 6.7±0.2$^{b}$ | 4.0±0.5$^{a}$ | 4.5±0.5$^{a}$ | 4.4±0.5$^{a}$ | |
| fatty odour | 0 days | 2.4±0.5 | 3.0±0.6 | 1.7±0.2 | 2.5±0.7 | |
| | 14 days | 3.4±0.6$^{b}$ | 1.9±0.2$^{a}$ | 2.4±0.3$^{ab}$ | 2.3±0.3$^{a}$ | |
| acid odour | 0 days | 1.5±0.2 | 1.0±0.3$^{A}$ | 1.2±0.2 | 1.0±0.3 | S*** |
| | 14 days | 1.8±0.4 | 2.3±0.4$^{B}$ | 2.0±0.4 | 1.7±0.4 | |
| sharp odour | 0 days | 1.5±0.3 | 2.0±0.2 | 0.9±0.2$^{A}$ | 1.9±0.3 | S* |
| | 14 days | 1.9±0.2 | 2.0±0.4 | 1.9±0.3$^{B}$ | 2.2±0.3 | |
| rancid odour | 0 days | 0.4±0.1 | 0.3±0.1$^{A}$ | 0.5±0.2 | 0.3±0.1$^{A}$ | T×S*** |
| | 14 days | 1.0±0.3$^{a}$ | 2.6±0.5$^{bB}$ | 1.1±0.3$^{a}$ | 0.9±0.3$^{aB}$ | |
| hardness | 0 days | 7.8±0.2$^{cB}$ | 7.4±0.4$^{bcB}$ | 6.2±0.6$^{aB}$ | 6.5±0.4$^{ab}$ | T×S*** |
| | 14 days | 5.3±0.4$^{cA}$ | 3.1±0.3$^{aA}$ | 4.1±0.3$^{bA}$ | 5.6±0.3$^{c}$ | |
| juiciness | 0 days | 6.1±0.6 | 4.1±0.6 | 5.8±0.6 | 5.6±0.6 | T*** |
| | 14 days | 5.1±0.3$^{b}$ | 3.2±0.3$^{a}$ | 4.9±0.4$^{b}$ | 5.1±0.4$^{b}$ | |
| colour | 0 days | 7.4±0.2$^{c}$ | 2.2±0.3$^{a}$ | 4.4±0.3$^{bA}$ | 4.3±0.2$^{b}$ | S*** T*** |
| | 14 days | 7.1±0.3$^{d}$ | 2.1±0.2$^{a}$ | 6.0±0.3$^{cB}$ | 4.2±0.3$^{b}$ | |
| smoked meat flavour | 0 days | 8.3±0.2$^{B}$ | 7.8±0.2$^{B}$ | 8.2±0.2 | 8.3±0.3$^{B}$ | T×S*** |
| | 14 days | 6.0±0.7$^{aA}$ | 6.8±0.2$^{abA}$ | 7.9±0.2$^{c}$ | 7.1±0.2$^{bcA}$ | |
| cured meat flavour | 0 days | 6.6±0.6$^{c}$ | 3.5±0.6$^{a}$ | 6.5±0.6$^{bc}$ | 4.9±0.6$^{ab}$ | T*** |
| | 14 days | 6.0±0.5$^{c}$ | 3.2±0.5$^{a}$ | 5.0±0.5$^{b}$ | 4.4±0.5$^{ab}$ | |
| salty flavour | 0 days | 4.2±0.6 | 4.5±0.6 | 3.3±0.7 | 4.0±0.8 | |
| | 14 days | 3.8±0.4$^{ab}$ | 3.5±0.4$^{a}$ | 4.8±0.3$^{b}$ | 3.4±0.3$^{a}$ | |
| fatty flavour | 0 days | 2.1±0.5 | 2.4±0.5 | 1.8±0.5 | 2.2±0.6 | |
| | 14 days | 1.6±0.2 | 2.0±0.2 | 2.1±0.2 | 1.8±0.2 | |

| | | | | | | | |
|---|---|---|---|---|---|---|---|
| acid flavour | 0 days | 1.6±0.4 | 1.3±0.4$^{A}$ | 1.3±0.5 | 1.5±0.6 | S* | |
| | 14 days | 1.8±0.3 | 2.5±0.4$^{B}$ | 2.2±0.4 | 2.2±0.4 | | |
| bitter flavour | 0 days | 0.7±0.1$^{A}$ | 0.6±0.1$^{A}$ | 1.0±0.2 | 0.7±0.2$^{A}$ | S*** | |
| | 14 days | 1.6±0.2$^{B}$ | 1.9±0.3$^{B}$ | 1.6±0.3 | 1.8±0.3$^{B}$ | | |
| sharp flavour | 0 days | 2.8±0.5 | 2.5±0.3 | 2.1±0.4 | 2.6±0.5 | | |
| | 14 days | 3.0±0.3 | 3.0±0.4 | 2.6±0.4 | 2.7±0.4 | | |
| rancid flavour | 0 days | 0.2±0.1$^{A}$ | 0.3±0.2$^{A}$ | 0.3±0.1$^{A}$ | 0.3±0.2$^{A}$ | S*** | |
| | 14 days | 1.0±0.3$^{aB}$ | 1.8±0.4$^{bB}$ | 1.0±0.3$^{aB}$ | 1.5±0.3$^{aB}$ | | |
| overall quality | 0 days | 8.9±0.2$^{bB}$ | 7.8±0.4$^{aB}$ | 8.8±0.2$^{bB}$ | 8.5±0.2$^{abB}$ | S*** | T*** |
| | 14 days | 8.0±0.2$^{bA}$ | 6.6±0.1$^{aA}$ | 8.1±0.2$^{2bA}$ | 7.1±0.2$^{aA}$ | | |

$^{a-c}$ Means with different letters differ significantly ($p < 0.05$) within the treatment. $^{A-B}$ Means with different letters differ significantly ($p < 0.05$) within time of storage. n=18. SE: standard error. *p*: significance of effects; treatment (t); time of storage (s); treatment × time of storage interaction (t×s); NS – not significant; *$p < 0.05$; ***$p < 0.001$. C - control cured treatment, S – salted treatment, LP - treatment with a salt and *Lactiplantibacillus plantarum* S21 at about $10^7$ CFU/g, AW - treatment with a salt and acid whey.
